# Oxidative Stress Predicts All-Cause Mortality in HIV-Infected Patients

**DOI:** 10.1371/journal.pone.0153456

**Published:** 2016-04-25

**Authors:** Mar Masiá, Sergio Padilla, Marta Fernández, Carmen Rodríguez, Ana Moreno, Jose A. Oteo, Antonio Antela, Santiago Moreno, Julia del Amo, Félix Gutiérrez, CoRIS, Biobanco

**Affiliations:** 1 Infectious Diseases Unit, Hospital General de Elche, Universidad Miguel Hernández, Alicante, Spain; 2 Infectious Diseases Research Laboratory, Hospital General de Elche, Alicante, Spain; 3 HIV/AIDS and Sexually Transmitted Diseases Clinic, Centro Sanitario Sandoval, Madrid, Spain; 4 Infectious Diseases Service, Hospital Ramón y Cajal, Instituto Ramón y Cajal de Investigación Sanitaria, Madrid, Spain; 5 Infectious Diseases Service, Hospital San Pedro de La Rioja, Logroño, Spain; 6 Infectious Diseases Unit, Hospital Clínico de Santiago, La Coruña, Spain; 7 Centro Nacional de Epidemiología, Instituto de Salud Carlos III, Madrid, Spain; FIOCRUZ, BRAZIL

## Abstract

**Objective:**

We aimed to assess whether oxidative stress is a predictor of mortality in HIV-infected patients.

**Methods:**

We conducted a nested case-control study in CoRIS, a contemporary, multicentre cohort of HIV-infected patients, antiretroviral-naïve at entry, launched in 2004. Cases were patients who died with available stored plasma samples collected. Two age and sex-matched controls for each case were selected. We measured F2-isoprostanes (F_2_-IsoPs) and malondialdehyde (MDA) plasma levels in the first blood sample obtained after cohort engagement.

**Results:**

54 cases and 93 controls were included. Median F_2_-IsoPs and MDA levels were significantly higher in cases than in controls. When adjustment was performed for age, HIV-transmission category, CD4 cell count and HIV viral load at cohort entry, and subclinical inflammation measured with highly-sensitive C-reactive protein (hsCRP), the association of F_2_-IsoPs with mortality remained significant (adjusted OR per 1 log_10_ increase, 2.34 [1.23–4.47], P = 0.009). The association of MDA with mortality was attenuated after adjustment: adjusted OR (95% CI) per 1 log_10_ increase, 2.05 [0.91–4.59], P = 0.080. Median hsCRP was also higher in cases, and it also proved to be an independent predictor of mortality in the adjusted analysis: OR (95% CI) per 1 log_10_ increase, 1.39 (1.01–1.91), P = 0.043; and OR (95% CI) per 1 log_10_ increase, 1.46 (1.07–1.99), P = 0.014, respectively, when adjustment included F_2_-IsoPs and MDA.

**Conclusion:**

Oxidative stress is a predictor of all-cause mortality in HIV-infected patients. For plasma F_2_-IsoPs, this association is independent of HIV-related factors and subclinical inflammation.

## Introduction

HIV infection is characterized by a progressive depletion of CD4^+^ T-cell populations and a state of chronic inflammation and immune activation [1, 2]. A related mechanism implicated in the pathogenesis of HIV disease and its complications is a pro-oxidative status associated with the infection and with antiretroviral therapy (ART) [1–5]. HIV induces the generation of reactive oxygen species (ROS) through the regulatory protein Tat and the envelope glycoprotein gp120 [3]. HIV-activated macrophages via TNF-α release, and activated polymorphonuclear leukocytes, also contribute to the generation and accumulation of ROS [4]. As a consequence, there is a deficiency in the antioxidant capacity of the organism, due in part to excessive consumption of antioxidant molecules in order to protect cells against ROS-induced damage [5], which contributes to further enhance the pro-oxidative status.

Numerous in vitro studies have linked oxidative stress with many aspects of HIV pathogenesis, including stimulation of HIV replication, numerical and functional impairment of CD4^+^ T cells, altered immune response, and toxicity of antiretrovirals [6–9]. It has also shown to play a central role in certain HIV-associated diseases, like HIV dementia [10]. Besides the HIV-related effects, oxidative stress has been associated with aging and with the development of several chronic diseases [11].

Increased oxidative stress biomarkers have been documented in HIV-infected and in AIDS patients compared to HIV-uninfected controls [5], and in patients receiving ART, with most studies being conducted in the era prior to currently recommended antiretroviral regimens [6, 9]. Despite the theoretical etiopathogenic role of oxidative stress in HIV disease, evidence from clinical studies is sparse. Oxidative stress biomarkers were found to be increased in patients with lipodystrophy and symptomatic hyperlactatemia in two cross sectional studies [12, 13], and to be associated with traditional and non-traditional cardiovascular risk factors [14], but did not predict peripheral neuropathy development in a longitudinal study of patients starting ART [15]. In addition, limited information is available about the association of oxidative stress with mortality in HIV patients.

F_2_-isoprostanes (F_2_-IsoPs) and malondialdehyde (MDA) are free radical-induced peroxidation products. Measurement of F_2_-IsoPs constitutes the most reliable approach to assess oxidative stress status in vivo [16]. MDA is also widely used as indicator of cellular injury [17]. We aimed to assess the role of plasma levels of F_2_-IsoPs and MDA as predictors of mortality in a contemporary cohort of HIV-infected patients.

## Methods

### Design, setting and study subjects

We conducted a nested case-control study in the ongoing open cohort of adults with HIV infection of the Spanish AIDS Research Network (CoRIS). This is a prospective, multicentre cohort of adult subjects with confirmed HIV infection, and naïve to ART at study entry. The cohort is linked to a centralized BioBank, where patients’ blood samples are processed, cryopreserved and stored. Participating centres are encouraged to obtain a first blood sample at engagement in the cohort, preferentially before starting ART, and follow-up samples preferentially annually, or at least biannually, thereafter. The BioBank has obtained the UNE-EN-ISO 9001:2008 Systems of Quality Management Requirements. Approval from each hospital’s Ethics Committee, and written informed consents from the patients, including the specific consent for the BioBank were obtained. Detailed description of CoRIS and the BioBank have been previously reported [18, 19].

Eligible subjects were all patients with available blood samples at the BioBank from cohort launching date (January 01, 2004) to administrative censoring date (October 31, 2010). Cases were all patients who died during the study period. For each case, two age (± 5 years) and sex individually-matched controls among those alive during the study period were randomly selected to increase the study efficiency. Due to insufficient plasma samples in selected controls, 15 of the cases could only be matched to one control each.

Date and causes of death were reported by the investigators to the coordinating center. Death due to an AIDS-defining event was defined as death attributable to a category C disease listed by the CDCs [20]. Death due to a non AIDS event was classified according to a revised version of the ‘Coding Death in HIV’ (CoDe) classification system [21].

The proportion of losses to follow-up in the cohort, defined as no information provided during the last year and no evidence of patients’ death, was below 20% [18].

### Variables, data sources and measurements

Blood samples were kindly provided by the BioBank. The first patients’ blood samples available after engagement in care were analysed. Malondialdehyde (MDA) was measured in plasma with a commercial high performance liquid chromatography (HPLC) kit (CHROMSYSTEMS, Gräfelfing/Germany). Plasma levels of 8-isoprostane were measured with a commercial EIA kit (Cayman Chemycal, Michigan 48108, USA). Both biomarkers have shown to be stable at -80°C for 6 months [22, 23]. Highly-sensitive C-reactive protein (hsCRP) was measured with a chemiluminescent immunometric assay (Immulite 2000, Siemens).

### Statistical analyses

Statistical analyses of the data were performed in R, version 3.0.2 (R Foundation for Statistical Computing, Vienna, Austria, URL http://www.R-project.org/). Median values were compared with the Mann-Whitney or Wilcoxon tests, where appropriate, and chi-square was used to compare proportions. F_2_-IsoPs, MDA and hsCRP biomarkers values were logarithmically transformed. Conditional logistic regression analysis incorporating the case-control matching factors was used to study the associations of baseline values of F_2_-IsoPs and MDA with all-cause mortality. Effects are quantified in terms of the odds ratio (OR) per 1 log_10_ change for each biomarker. Adjusted analyses controlled for age, injection drug use (IDU) versus other HIV-transmission categories, CD4 cell count and HIV-RNA at cohort entry, and log_10_ hsCRP.

## Results

Fifty four patients who died during the study period and 93 controls with available stored serum samples were identified. The causes of death were AIDS conditions (49.1%), non-AIDS events (38.2%), and unknown (12.7%). Most patients (91.9%) were male, and median (interquartile range, IQR) age at cohort entry was 46.7 (40.1–51.1) years. Other baseline characteristics of cases and controls are shown in Table 1.

**Table 1:** Baseline characteristics of the patients.

Variable	All		Cases		Controls		*p* value^¶^
**Patients**, no.	**147**		54		93		-
**Female**, no. (%)	**12**	**(8.1)**	5	(9.2)	7	(7.5)	0.955
**Age at cohort entry**, median years (IQR)	**46.7**	**(40.1–51.1)**	47.8	(42.1–52.4)	46.1	(40.0–51.0)	0.377
**HIV transmission groups**, no. (%)							0.435
IDU	**24**	**(16.3)**	11	(20.4)	13	(14.0)	
Non-IDU	**123**	**(83.7)**	43	(79.6)	80	(86.0)	
Education level*, no. (%)							0.005
Low	**68**	**(46.2)**	32	(59.2)	36	(38.7)	
Medium	**33**	**(22.4)**	7	(12.9)	26	(27.9)	
High	**30**	**(20.4)**	6	(11.1)	24	(25.8)	
Unknown	**16**	**(10.8)**	9	(16.6)	7	(7.5)	
**Country of origin**, no. (%)							0.122
Spain	**143**	**(97.3)**	54	(100)	89	(95.7)	
Other	**4**^#^	**(2.7)**	0	(0)	4	(4.3)	
**AIDS diagnosis at cohort entry,** no. (%)	**21**	**(14.2)**	13	(24.0)	8	(8.6)	0.012
**CD4 (cells/μL) at cohort entry**^$^, no. (IQR)	**252**	**(69–475)**	86	(29–247)	360	(160–555)	0.001
**Plasma HIV viral load (log**_**10**_, **copies/mL) at cohort entry**^$^, median (IQR)	**4.43**	**(3.56–5.25)**	4.77	(2.73–5.37)	4.39	(3.68–5.16)	0.168
**Patients with virological suppression**^&^, no. (%)	**28**	**(19.0)**	12	(22.2)	16	(17.2)	0.596
**Patients on treatment,** no. (%),	**34**	**(23.1)**	18	(33.3)	16	(17.2)	0.042
**Hepatitis C virus coinfection**, no. (%)	**30**	**(20.4)**	15	(27.7)	15	(16.1)	0.028
**Follow-up**¥, median years (IQR)	**2.1**	**(0.70–4.48)**	0.79	(0.25–2.39)	2.98	(1.43–5.07)	0.001

IQR, interquartile range; IDU, injection drug users.

^¶^*p* value between cases and control groups: Wilcoxon or Chi-squared tests were used where appropriate.

* Education level definition was based on the level of education completed at cohort entry, and subjects were classified into three levels: low, individuals with no education or with primary education; medium, individuals who completed secondary education; and high, individuals who completed university education.

^**$**^Median (IQR) difference of days between cohort inclusion to CD4/viral load measurements was 0 (0–5) days

^&^Virological suppression was defined as an HIV RNA < 200 copies/ml in the nearest determination to the biomarkers measurement.

^#^The four patients were born in Latin America.

^¥^, Years from cohort inclusion to which happened first: death, lost of follow-up or administrative censoring

### Biomarkers of oxidative stress and subclinical inflammation

Median (interquartile range, [IQR]) F_2_-IsoPs and MDA levels are shown in Table 1. The majority of patients were ART naive when the first blood sample was collected, although 23% of patients had initiated ART, and 19% patients were virologically suppressed (HIV RNA < 200 copies/ml) at the time of the first available sample at the BioBank. Median (IQR) number of days from cohort enrollment to first blood sample used in the biomarkers determination was 23 (3–166) days; 22 (5.5–159) days for cases and 23 (2.5–179) days for controls, P = 0.082.

**Fig 1:**
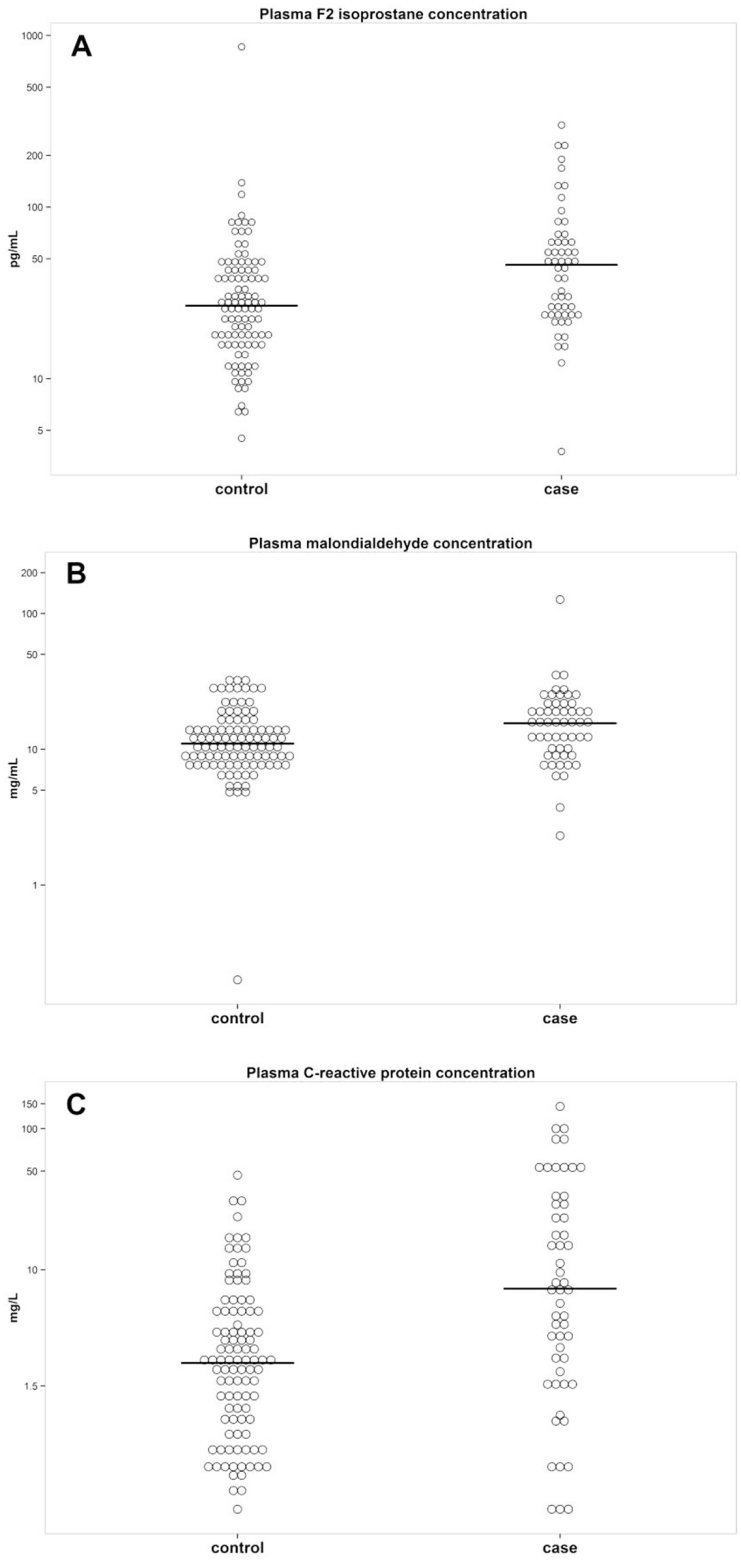
Plasma levels of F2-isoprostanes, malondialdehyde and C-reactive protein in cases and controls.

Median (IQR) levels of F_2_-IsoPs and MDA were higher in patients who died as compared to their matched controls: 46.20 (24.06, 64.68) pg/mL vs 26.64 (17.32–42.40) pg/mL, respectively, for F_2_-IsoPs, P = 0.001; and 15.56 (9.84, 20.49) mg/mL vs 11.01 (8.16, 14.41) mg/mL, respectively, for MDA, P = 0.008 (Table 1, Fig 1).

**Fig 2:**
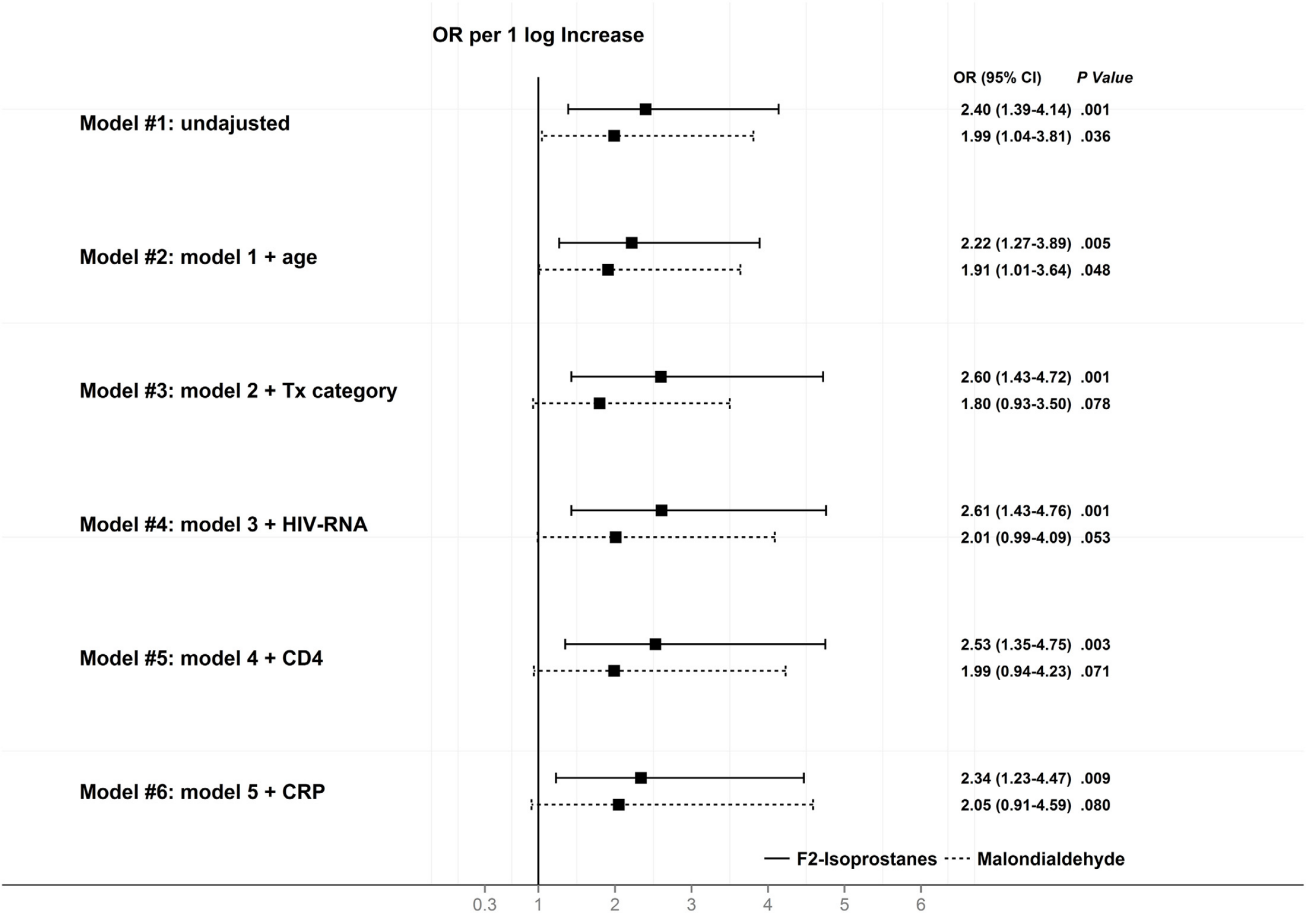
Unadjusted and adjusted by conditional logistic regression odds ratios for death for plasma F2-isoprostanes and malondialdehyde levels.

The odds ratio (OR) for death per 1 logarithm increase in the biomarkers levels in the unadjusted and adjusted analyses are shown in Fig 2.

When adjustment was performed for age, HIV transmission category (IDU versus non-IDU), CD4 cell count and HIV-RNA at cohort entry, the association of F_2_-IsoPs with mortality remained significant (adjusted OR [95% CI] per 1 log_10_ increase for F_2_-IsoPs level was 2.53 [1.35–4.75], P = 0.003), and there was a mild attenuation of the relationship of mortality with MDA, with a close to significant association (adjusted OR [95% CI] per 1 log_10_ increase for MDA was 1.99 [0.94–4.23], P = 0.071). The model was not adjusted for hepatitis C virus, because of collinearity with IDU. Adding hsCRP to the adjusted model did not alter significantly the relationship of the biomarkers with mortality: adjusted OR (95% CI) per 1 log_10_ increase for F_2_-IsoPs level was 2.34 (1.23–4.47), P = 0.009; adjusted OR (95% CI) per 1 log_10_ increase for MDA was 2.05 (0.91–4.59), P = 0.080.

Median levels of hsCRP were also higher in cases than in controls (Fig 1). When adjustment was performed for age, HIV transmission category, CD4 cell count and HIV-RNA at cohort entry, and the oxidative stress biomarkers levels, hsCRP showed to be an independent predictor of mortality: OR (95% CI) per 1 log_10_ increase, 1.39 (1.01–1.91), P = 0.043, and OR (95% CI) per 1 log_10_ increase, 1.46 (1.07–1.99), P = 0.014, respectively, when adjustment included F_2_-IsoPs and MDA.

## Discussion

The oxidative stress biomarkers F_2_-IsoPs and MDA predict all-cause mortality in HIV-infected patients. For F_2_-IsoPs, this association is independent of the HIV transmission category, CD4 cell count, HIV viral load, and subclinical inflammation measured with hsCRP.

This is, as far as we know, the first cohort study to show an independent association of oxidative stress with death in HIV-infected patients. Our results indicate that oxidative stress constitutes an additional predictor of mortality, independent of established HIV-associated predictors such as CD4 cell count and HIV viral load, and also of inflammation. Likewise, the association was independent of the HIV transmission group. Injection drug use has been linked with increased all-cause mortality in the HIV population, including AIDS and non-AIDS events [24], and with increased oxidative stress in animal models and in clinical studies in HIV patients [25]. Adjusting for IDU did not alter the relationship of oxidative stress with mortality in our cohort.

Oxidative stress has been implicated in the pathogenesis of HIV disease, and it’s considered to play an important role in the progression from the asymptomatic stage to the development of AIDS [26]. Reactive oxygen species activate the NF-κ B transcription factor, that induces the expression and replication of HIV in human T cells [27]. NF-κ B also acts as a transcription factor for many inflammatory cytokines, like TNF-α, which further activates HIV replication [28]. Oxidative stress has shown to induce as well an abnormal immune response through functional impairment of T cells and DNA damage and apoptosis of CD4^+^ T lymphocytes, leading to CD4^+^ cell depletion [1, 8]. AIDS was a frequent cause of mortality in our cohort, which supports the relationship of oxidative stress with advanced disease and with disease progression. Noteworthy, an important proportion of these patients died relatively soon after cohort entry, probably representing delayed diagnoses. Apart from AIDS conditions, a high proportion of patients died as a consequence of non-AIDS events, as we had previously stated [29]. Oxidative stress has been implicated in cellular senescence and aging, and in the development of several chronic diseases including cancer, non-alcoholic liver disease, neurodegenerative disorders, or cardiovascular disease, among others [10, 30–32]. In animal studies, increased oxidative stress has been linked with shorter life expectancy [33]. However, data about the relationship of oxidative stress biomarkers with mortality in humans are limited. Recently, an association has been described with all-cause mortality in HIV-uninfected older adults [34]. Studies on specific oxidative biomarkers are limited to circumscribed clinical scenarios [35, 36].

We found that the relationship of oxidative stress biomarkers with mortality was independent of the HIV viral load at the time of engagement to care in the cohort. Because the proportion of patients under ART at study baseline was low, we could not separately evaluate the role of oxidative stress as a mortality predictor in virologically-suppressed patients. Available information to date regarding the influence of ART on oxidative stress is contradictory. While an improvement has been described with ART [8], there are a high number of studies linking ART with the induction of oxidative stress [6, 9]. Most of the main antiretroviral families, and even particular antiretrovirals, have been implicate; however, clinical studies addressing this unfavourable association generally included older ART regimens, frequently based on thymidine analogues, which might have contributed through mitochondrial toxicity to a pro-oxidizing status. The influence of currently used antiretroviral regimens on the oxidative balance remains to be defined. Alternative measures to ART, including supplementation with micronutrients containing antioxidants, have been explored in the HIV population, with benefits shown in morbi-mortality in African pregnant women and children [37]. A large randomized controlled clinical trial is currently being conducted comparing high-dose micronutrient and anti-oxidant supplementation versus recommended daily allowance vitamins to slow HIV immune deficiency progression in ART-naïve people with HIV infection [38].

The association of F_2_-IsoPs, and more marginally of MDA levels, with mortality was also independent of subclinical inflammation measured with hsCRP levels at study entry. Moreover, our study found that, in addition to the oxidative stress biomarkers, hsCRP is an independent predictor of mortality in HIV-infected patients as well. This supports the pathogenic role of inflammation in the development of complications and fatal outcome of the patients, as previously stated [39, 40]. Our results also suggest that F_2_-IsoPs, and to a lesser extent MDA levels, may point to a pathogenic pathway acting beyond inflammation that leads to tissue damage and death. Reactive oxygen species have been associated with aging and with lower life span by inducing structural damage on various macromolecules although, more recently, a functional impairment of the redox-regulated signaling mechanisms as a consequence of a pro-oxidizing shift in the cells has been postulated as a more likely hypothesis [41]. In addition, increased oxidative stress has been associated with accelerated telomere shortening [42], a mechanism underlying cellular aging and contributing to mortality. In HIV-infected patients, an inverse relationship has been described between telomere length and progression of immunosuppression [43] or immunological recovery despite a successful virological response [44].

A limitation of the study is the insufficient number of follow-up samples to verify that results were equivalent to those obtained with the first available samples. Another limitation consists on the potential bias introduced by the patients who were lost to follow-up, in whom the vital status is unknown. The limited number of patients precluded us from adjusting for additional relevant factors, including cardiovascular risk factors, and this could result in an over or underestimation of results. By contrast, information about covariates of interest was equally available for cases and controls. Some of the limitations inherent to case-control designs, such as the ascertainment of exposure, have been overcome given the availability of a biobank linked to the cohort. Unfortunately, due to low numbers some of our estimates are imprecise but are, nevertheless, extremely consistent. The association of hsCRP with mortality is also consistent with the results found in large cohorts [39, 40].

In conclusion, oxidative stress predicts all-cause mortality in HIV-infected patients. For plasma F_2_-IsoPs, this association is independent of HIV-related variables and subclinical inflammation. Our results support the pathogenic role of oxidative stress in HIV disease identified in experimental studies, and may suggest additional measures to ART to improve health status of HIV-infected patients.
